# Enhanced chlorine evolution from dimensionally stable anode by heterojunction with Ti and Bi based mixed metal oxide layers prepared from nanoparticle slurry

**DOI:** 10.1016/j.jcat.2020.04.009

**Published:** 2020-09

**Authors:** Sukhwa Hong, Tai-kyu Lee, Michael R. Hoffmann, Kangwoo Cho

**Affiliations:** a Division of Environmental Science and Engineering, Pohang University of Science and Technology (POSTECH), Pohang 790-784, Republic of Korea; b Nanopac Co., Ltd. 673 Hwasan-Ri, Cheoin-Gu, Yongin-Si, Gyeonggi-Do, Republic of Korea; c Linde+Robinson Laboratories, California Institute of Technology, 1200 E. California Blvd., Pasadena, CA 91125, USA; d Institute for Convergence Research and Education in Advanced Technology (I-CREATE), Yonsei University International Campus, Incheon, Republic of Korea

**Keywords:** Heterojunction anode, Reactive chlorine species, Wastewater treatment, Dimensionally stable anode, TiO_2_

## Abstract

This study reports enhanced current (CE_RCS_) and energy efficiency (EE_RCS_) of reactive chlorine species (RCS) generation on Ir_7_Ta_3_O_y_ anode by Ti/Bi mixed metal oxide heterojunction layers despite reductions in pseudo-capacitance and film conductivity. In potentiostatic electrolysis of 50 mM NaCl solutions, dramatic improvement (0.61 mmol cm^−2^ hr^−1^ at 2.5 V NHE) was noted by simple coating of thin (~2 μm) TiO_2_ layer from ball-milled TiO_2_ nanoparticle (80–100 nm) suspension, even with moderate elevation in voltammetric wave. Decoration of Bi_2_O_3_ particles (1 – 2 μm) showed limited or adverse effects for RCS generation and stability. However, Bi-doped TiO_2_ layers prepared from polyol-mediated or co-precipitation methods marked the highest CE_RCS_ (~100%) and EE_RCS_ (8.16 mmol Wh^−1^ at 2.5 V NHE) by increased mixing level and effective shift in surface charge. Surface ·OH exclusively mediated the RCS generation whose further transformation to higher oxide could be restrained by the heterojunction layer.

## Highlights

• TiO_2_ nanoparticle over-layer accelerate reactive chlorine generation from Ir_7_Ta_3_O_y_.
• Uniform doping of Bi on TiO_2_ further elevate current and energy efficiency.
• Surface ·OH exclusively mediate the reactive chlorine generation.
• Ratio of ·OH to higher oxide primarily determine the current efficiency.

## Graphical abstract

**Figure:**
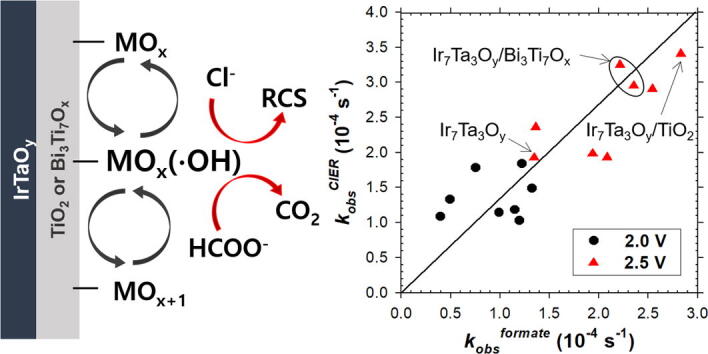

## 1 Introduction

Chlorine (Cl_2_) is a widely utilized industrial reagent for polymer synthesis globally. In chemical industry, chlor-alkali process would be one of the most commercialized methods to electrolyze concentrated NaCl solutions for generation of Cl_2_, H_2_, and caustic soda (NaOH). Dimensionally stable anodes (DSAs), based on precious metal oxides such as RuO_2_ and IrO_2_, have been most often used for these purposes owing to the supreme activity and stability [1], [2], [3]. Variable secondary components have been explored primarily for stability enhancement, to come up with the mixed metal oxide anodes, Ir_7_Ta_3_O_x_ and Ru_3_Ti_7_O_x_ as the optimal DSAs compositions for many decades [1], [3], [4].

Aqueous Cl_2_ (commonly noted as free chlorine) is also a famous oxidant for drinking water disinfection and, recently, electrolytic reactive chlorine species (RCS) has been utilized for on-site treatment of saline wastewater as well [5]. Owing to the value consuming nature of environmental technologies, however, the scarcity of Ir and Ru components have substantially constrained a large scale application of the electrochemical chlorination for water treatment. In addition, the DSAs active for chlorine evolution reaction (ClER, E°(Cl_2_/Cl^−^) = 1.36 V NHE) also effectively catalyze oxygen evolution reaction (OER, E°(O_2_/H_2_O) = 1.23 V NHE) [6], a parasitic side reaction with respect to the water treatment. A sufficient anodic potential bias on hydrous metal oxide (>MO_x_) would initiate the formation of surface hydroxyl radical (>MO_x_(∙OH), Eq. (1)) whose further oxidation could lead to higher oxide (>MO_x+1_, *via* Eq. (2)) formation. It has been widely accepted that the overall adsorption energy of oxygen to metal (M−O bond strength) is known to be the principal factor of OER activity mostly *via* Eq. (4) [7], [8], [9], [10].

(1) > MO_x_ + H_2_O → > MO_x_(∙OH) + H^+^ + e^−^

(2) > MO_x_(∙OH) → > MO_x+1_ + H^+^ + e^−^

(3) > MO_x_(∙OH) → > MO_x_ + 1/2 O_2_ + H^+^ + e^−^

(4) > MO_x+1_ → > MO_x_ + 1/2 O_2_

In a presence of chloride ions, parallel ClER mechanism on metal oxide electrocatalysts can be described by Eqs. (5), (6), where the ClER could share active sites with the competing OER [6], [8]; *i.e*., MO_x_(∙OH) and MO_x+1_ mediate the ClER as well.

(5) > MO_x_(∙OH) + Cl^−^ → > MO_x_ + 1/2 Cl_2_ + OH ^−^

(6) > MO_x+1_ + Cl^−^ → > MO_x_ + ClO^−^

Therefore, the selectivity towards ClER should be an important consideration for an energy-efficient electrochemical RCS mediated water treatment, especially considering limited Cl^−^ concentration (<50 mM) in (waste)water electrolyte [11].

Specific group of metal oxides such as PbO_2_, SnO_2_, and TiO_2_ could be characterized by a weak bond strength between metal and active oxygen to predominantly exist in the >MO(·OH) form which would prefer ClER due to stronger oxidation power than MO_x+1_. In particular, TiO_2_ is non-toxic and widely-used earth-abundant catalysts to provide > Ti(·OH) under an anodic potential with high surface density. Nevertheless, relatively large kinetic barrier for the water discharge has limited the utilization of TiO_2_ as an electrocatalyst. We previously proposed a heterojunction architecture with coating TiO_2_ layers with or without mixing Bi (Bi_x_Ti_1−x_O_z_) on top of Ir_7_Ta_3_O_y_ DSA for an enhanced RCS generation in dilute aqueous solutions [11]. In these configurations, conductor-like nature of IrTaO_y_ could serve as an ohmic contact to Ti substrate [11], [12], while surface hydrous TiO_2_ (>Ti(OH^–^)) provided elevated quasi-stationary concentration of >MO_x_(∙OH). In spite of limited consideration for limited active sites and different kinetic parameters to predict selectivity in parallel reactions, density functional theory (DFT) calculations on model RuO_2_/TiO_2_ architecture [13] further suggested that the TiO_2_ heterojunction layer could shift locations in volcano plots of OER and ClER, which in-turn influences the ClER selectivity. In addition, mixing Bi in the outer layer was evinced to increase electrostatic sorption of anions (Cl^−^). In our earlier contribution, nevertheless, the peroxo-route for aqueous Ti-glycolate complex and solution casting of the Bi_x_Ti_1−x_O_z_ layer were rather dangerous and labor consuming.

This study reports simplified preparation methods for coating TiO_2_ based heterojunction layers (on Ir_7_Ta_3_O_y_ DSA) which was either decorated by Bi_2_O_3_ micro-particles or mixed with Bi^3+^. In particular, employing nanoparticle slurry precursors was expected not only to augment the catalytic edge sites [3], but also to allow an effective passivation owing to greater viscosity than aqueous precursor [14]. In dilute (50 mM) NaCl solutions, the activity and selectivity for ClER were comparatively evaluated for the heterojunction anodes with variable outer layer loading (thickness) and mixing levels of Bi. In addition, the roles of >MO_x_(∙OH) and >MO_x+1_ on the RCS generation were interrogated by correlation with formate ion degradation.

## 2 Experimental section

### 2.1 Preparation and characterization of the anodes

All chemical reagents were analytical grade and obtained from Sigma-Aldrich or Daejung chemicals to be used without further purification. Solutions were prepared with Millipore-Q water (Millipore) with a specific resistivity of 18 MΩ cm^−1^. For pretreatment of the anode base, Ti foils (99.5% Alfa-Aesar) were cleaned with SiC paper, sonicated in organic solvent solutions (equivolumic deionized water, ethanol, and acetone) for 0.5 h, and immersed in 10 wt% oxalic acid for 50 min at 80 °C. The IrTaO_y_ layer was prepared by soaking the Ti substrate (3 × 2 cm^2^, distance = 5 mm) into solutions with 73 mM H_2_IrCl_6_, and 27 mM TaCl_5_. 4 N HCl and equivolumic ethanol/isopropanol solutions were compared as solvents. The loaded precursor was sequentially dried at 80 °C and annealed at 525 °C for 10 min, which was repeated five times before final annealing at 525 °C for 1 h. For preparation of outer heterojunction layers, three different types of precursor were prepared as follows. A) P90 TiO_2_ (Evonik Industries) and Bi_2_O_3_ particles separately ball-milled (Ultra Apex mill) in ethanol to have particle size range of 80–100 nm and 1–2 μm, respectively. The TiO_2_ nanoparticles (3.8 wt%) were dispersed in ethanol/terpineol (16:19 wt%) solution with added ethyl cellulose binder (2.2 wt%). Bi_2_O_3_ micro-particles were added with variable Bi to Ti ratios (0:10, 1:9, and 3:7). B) 6 mL titanium butoxide was added to 150 mL ethylene glycol to be stirred for 8 h in room temperature. The mixture was poured into 510 mL acetone and 8 mL of deionized (DI) water was added before vigorous stirring for 1 h. Resulting precipitate was collected using a centrifuge (6000 rpm for 4 min), washed with ethanol 5 times, and re-dispersed in ethanol with a given concentration of bismuth citrate. C) titanium tetraisopropoxide (1.25 mL) in ethanol (25 mL) was added to 8 mM Bi(NO_3_)_3_ solution (pH 1.5 adjusted by acetic acid) by dropwise and the mixture was stirred overnight. Resulting precipitate was collected (11000 rpm for 10 min), washed with DI water 5 times, and re-dispersed in DI water. These precursors were sprayed on the IrTaO_y_ layer and annealed at 450 °C for 30 min and the coating procedure was performed for 3 times. In this study, heterojunction anodes from precursor A were denoted as (Bi_2_O_3_)_x_(TiO_2_)_1−x_ (x = 0, 0.1, and 0.3), while the outer layers with low mass loading (L) were prepared without repetition to compare the performance with analogues with high mass loading (H). The anodes from precursor B and C with nominal Bi to Ti ratio of 3:7 were named as Bi_3_Ti_7_O_x_-1 and Bi_3_Ti_7_O_x_-2, respectively, to describe greater mixing level of Bi.

The surface morphologies were observed by using a ZEISS 1550VP field emission scanning electron microscope (SEM) and elemental compositions were estimated using an Oxford X-Max SDD X-ray energy-dispersive spectrometer (EDS) system. The EDS analysis was performed either in a point-and-identification mode for 10 arbitrary sites or in mapping mode. The surface crystallography was assessed by X-ray diffraction (XRD) using an X’pert MD (Panalytical) diffractometer with Cu−K radiation.

### 2.2 Electroanalysis.

The electrochemical activities of (Bi_2_O_3_)_x_(TiO_2_)_1−x_ and Bi_3_Ti_7_O_x_-1/2 heterojunction anodes were assessed using linear sweep voltammetry (LSV) and cyclic voltammetry (CV). In a single compartment cell (working volume = 60 mL), each anode was parallel matched with AISI 304 stainless steel cathode (distance = 5 mm) with active geometric surface area of 3 × 2 cm^2^. Ag/AgCl/sat. KCl reference electrode (BASI Inc.) was located 3 mm away from the anode center. The three electrodes configuration was connected to a potentiostat (SP-50, Bio-Logic) to control anodic potential (*E_a_*) in normal hydrogen electrode (NHE) scale (*E_a_* (NHE) = *E_a_* (Ag/AgCl) + 0.197 V). The CV and LSV data were recorded with *E_a_* ranges of 0.2–1.0 V NHE (scan rate of 20 mV s^−1^) and of 0.8–2.0 V NHE (scan rate of 5 mV s^−1^), respectively. Before all electrochemical experiments, open circuit potentials were measured for 30 min and ohmic resistances were measured by current interruption method at 200 mA current bias.

### 2.3 Potentiostatic Electrolysis.

The efficiency of RCS generation was evaluated during potentiostatic electrolysis of 50 mM NaCl solutions at variable *E_a_* values (2.0, 2.5, and 3.0 V NHE). The evolution of [RCS] was measured by DPD (N, N-diethyl-p-phenylenediamine) reagents 3 times at 2 min intervals and, during this period, further oxidation of RCS to ClO_3_^−^ or ClO_4_^−^ could be negligible [15]. The specific rate (SR), current (CE) and energy efficiency (EE) of the RCS generation were estimated by the following equations (Eqs. (7)–(9)).

(7) $${SR}_{RCS}\;{(mmol\;cm}^{-2}\;)=\frac{Vd[{Cl}_{DPD}]}{Adt}$$

(8) $${CE}_{RCS}\;\left(\%\right)=\frac{2VFd[{\mathrm{Cl}}_{\mathrm{DPD}}]}{\mathrm{Idt}}$$

(9) $${EE}_{RCS}\;\left({mmol\;Wh}^{-1}\right)=\frac{\mathrm{Vd}[{\mathrm{Cl}}_{\mathrm{DPD}}]}{\mathrm{EIdt}}$$

where *V* is electrolyte volume (0.06 L), *F* is Faraday constant (96485.3 C mol^−1^), [Cl_DPD_] is the concentration of RCS (M), *t* is electrolysis time (s), *A* is electrode surface area (cm^2^), *E* is cell voltage (V) and *I* is current (A).

In order to assess the roles of >MO_x_(·OH) on RCS generation, formate ion was employed as the ·OH probe compound. The potentiostatic electrolysis of 50 mM NaCOOH solutions was performed for 2 h at *E_a_* 2.0 or 2.5 V NHE. The [HCOO^–^] of samples were periodically quantified by ion chromatography (IC, Dionex, USA) with an anion-exchange column (Ionpac AS 19).

## 3 Results and discussion

### 3.1 Physico-chemical characteristics of (Bi_2_O_3_)_x_(TiO_2_)_1−x_ heterojunction layers

For the heterojunction electrodes, the underlying IrTaO_y_ layer was synthesized using an organic solvent (ethanol/isopropanol solutions), due to more uniform surface coverage and moderately greater electrochemically active surface area (ECSA) than analogous from hydrochloric acid solvents (Figure S1 and S2). SEM images in Figure S3 show the horizontal surface morphology of the (Bi_2_O_3_)_x_(TiO_2_)_1−x_ heterojunction anodes with variable molar ratios of Ti to Bi and catalysts loading (thickness). Cross-sectional SEM images estimated the average thickness of (TiO_2_)-L and (TiO_2_)-H to be *ca.* 2 and 5 µm, respectively. (TiO_2_)-H and -L showed marginal numbers of crack and pinhole that are typically observed for thermally decomposed (mixed) metal oxide electrodes. Since P60 TiO_2_ nanoparticles already underwent annealing, further thermal expansion of the crystalline TiO_2_ particles could be limited, while the aggregation of nanoparticles would alleviate the topological distortion. For samples with added Bi_2_O_3_ particles in precursor solutions, discrete islands of bismuth oxide scattered on the surface were noted. EDS mapping in Figure S3g-h clearly identified the immobilized particles (diameter ~ 1 μm) to be Bi_2_O_3_.

**Fig. 1:**
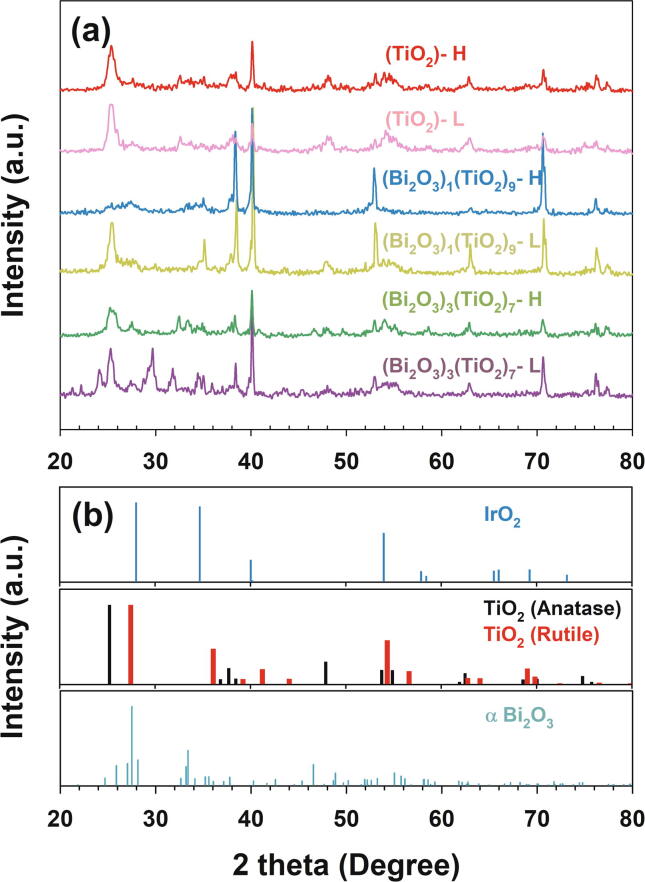
(a) X-ray diffraction patterns of (a) IrTaO_y_/(Bi_2_O_3_)_x_(TiO_2_)_1−x_ heterojunction anodes (x = 0, 0.1, and 0.3, mass loading = high (H) and low (L)), referenced with (b) IrO_2_, TiO_2_ (anatase, rutile) and Bi_2_O_3_.

In the absence of Bi mixing ((TiO_2_)-L and -H), the primary XRD peaks in Fig. 1 (individual patterns in Figure S4) commonly involved standard patterns of anatase TiO_2_ (2θ = 25.2, 37.7, and 47.9°) and less prominently of rutile TiO_2_ (2θ = 27.5, 36.1, and 54.4°), being in compatible with the composition of precursory TiO_2_ particles (92:8 wt% of anatase:rutile for P60). A phase transition during the thermal annealing at 450 °C would be limited since transformation into the rutile structure is known to occur at a temperature normally exceeding 600 °C [16]. Negligible characteristic pattern of IrO_2_ (2θ = 34.7, 40, 54, and 69.3°) on surface of these anodes indicated relatively thorough covering even with single spray coating. In comparison, (Bi_2_O_3_)_3_(TiO_2_)_7_–L electrodes showed clear patterns of standard α-Bi_2_O_3_ (2θ = 27.5, 33.2, and 46.5°), while the repeated coating of the Bi/Ti mixed precursor (-H) alleviated the signal from Bi_2_O_3_ buried in TiO_2_ matrix. The addition of Bi_2_O_3_ particles to the precursor gave negligible changes in diffraction peak positions of TiO_2_, indicating limited thermal diffusion between the separate crystalline lattice structures (TiO_2_ and Bi_2_O_3_) [17]. Therefore, each metal oxide phase was expected to maintain the intrinsic physicochemical properties with minimal doping effects; thus, the outer heterojunction layers could be denoted as (Bi_2_O_3_)_x_(TiO_2_)_1−x_. In the presence of Bi_2_O_3_ particles, signals from underlying IrO_2_ (peak near 35°) and Ti substrate (peak near 40°) were invigorated. Considering that the XRD analysis was performed after the electrochemical analyses described hereinafter, these observations suggest the instability of bismuth oxide in anodic environment (*vide-infra*).

### 3.2 Voltammetric responses of IrTaO_y_/(Bi_2_O_3_)_x_(TiO_2_)_1−x_ heterojunction anodes

**Fig. 2:**
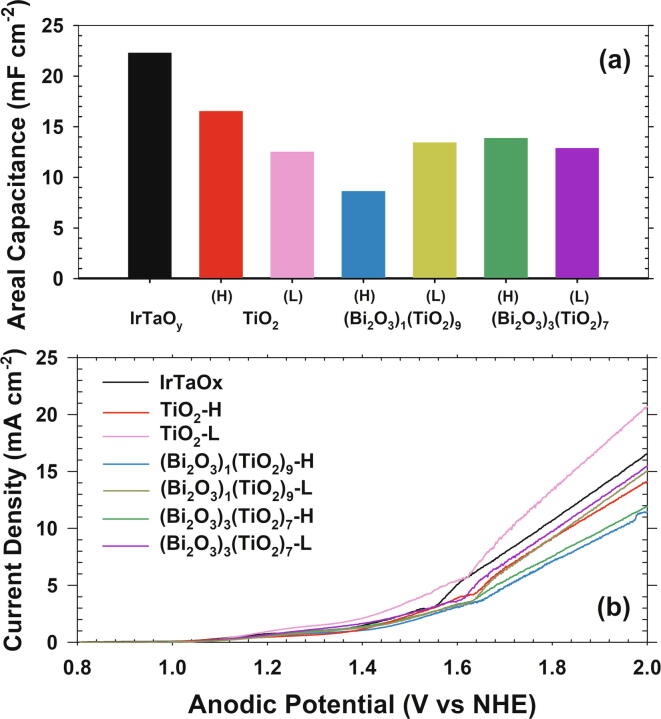
(a) Areal capacitance measured by cyclic voltammetry (scan range: 0.2–1.0 V NHE, scan rate: 20 mV s^−1^) and (b) linear sweep voltammograms (scan range: 0.8–2.0 V NHE, scan rate: 5 mV s^−1^) of Ir_7_Ta_3_O_y_/(Bi_2_O_3_)_x_(TiO_2_)_1−x_ heterojunction anodes (x = 0, 0.1, and 0.3, mass loading = high (H) and low (L)); electrolyte: 50 mM NaCl (pH 7), cathode: stainless steel, geometric surface area: 3 × 2 cm^2^. Data for Ir_7_Ta_3_O_y_ anode are shown as references.

Areal capacitance from CV within the potential window of water splitting has often been utilized to estimate the ECSA [18], [19]. Fig. 2a (raw CV data in Figure S5) illustrates the areal capacitance measured under anodic potential between 0.2 and 1.0 V NHE in 50 mM NaCl solutions (circum-neutral pH). The highest areal capacitance (22 mF cm^−2^) of IrTaO_y_ was reduced by about 30% upon addition of surface TiO_2_ layers. Mixing Bi_2_O_3_ particles on surface insignificantly affected or even further decreased the areal capacitance. Within the potential scan range, oxidative transformations of hydrous > TiOH or > BiOH (*e.g*., to oxyhydroxide or higher oxide) could be limited to especially due to the fully oxidized Ti^4+^. Nevertheless, considering rather thorough surface passivation by TiO_2_ layers (at least for IrTaO_y_/TiO_2_ anodes as shown in Fig. 1), the electrochemical activity for the heterojunction anodes would be ascribed to a thermal diffusion of Ir components into the upper layer (*vide infra*) [11].

Linear sweep voltammetry (scan range: 0.8 - 2.0 V NHE, scan rate: 5 mV s^−1^) in 50 mM NaCl solutions (Fig. 2b) estimated the anodic potential at 10 mA cm^−2^ in the order of TiO_2_-L < IrTaO_y_ < TiO_2_-H ~ (Bi_2_O_3_)_1_(TiO_2_)_9_-L ~ (Bi_2_O_3_)_3_(TiO_2_)_7_-L < (Bi_2_O_3_)_1_(TiO_2_)_9_-H ~ (Bi_2_O_3_)_3_(TiO_2_)_7_-H (Table 1). In these experimental conditions, the current generation would be ascribed to parallel OER and ClER. Electrical conductivity of semiconductor electrocatalysts should be another consideration especially for thick catalyst film. To this end, the outer heterojunction layer mostly lowered the current generation due to the inherently poor electrical conductivity of TiO_2_ and Bi_2_O_3_. More pronounced current decline with included Bi_2_O_3_ particles, particularly with higher loading ((Bi_2_O_3_)_1_(TiO_2_)_9_-H and (Bi_2_O_3_)_3_(TiO_2_)_7_-H), agreed with the concurrent increase in ohmic resistance measured by a current interruption method. The current interruption would collectively evaluate the electrolyte resistance of ion migration, polarization resistance and resistance across the catalytic film, often described as resistances in series in an equivalent circuit [20]. Since the Bi_2_O_3_ particles were speculated to be anchored within TiO_2_ matrix without a direct contact with IrTaO_y_ layer, (Bi_2_O_3_)_x_(TiO_2_)_1−x_ anodes would suffer from invigorated resistance across the multiple junctions (IrTaO_y_/TiO_2_/Bi_2_O_3_).

**Table 1:** Anodic potential at 10 mA cm^−2^ in linear sweep voltammetry (scan range: 0.8–2.0 V NHE, scan rate: 5 mV s^−1^) and solution resistance (R_s_) measured by current interruption (200 mA); electrolyte: 50 mM NaCl (pH 7), cathode: stainless steel, geometric surface area: 3 × 2 cm^2^.

Electrode	*E_a_* (V, *j* = 10 mA cm^−2^)	*R_s_* from current interruption (Ω)
IrTaO_x_	1.777	4.115
TiO_2_-H	1.834	4.090
TiO_2_-L	1.716	3.414
(Bi_2_O_3_)_1_(TiO_2_)_9_-H	1.940	4.846
(Bi_2_O_3_)_1_(TiO_2_)_9_-L	1.829	3.676
(Bi_2_O_3_)_3_(TiO_2_)_7_-H	1.912	5.427
(Bi_2_O_3_)_3_(TiO_2_)_7_-L	1.809	4.054
Bi_3_Ti_7_O_x_-1	1.862	4.455
Bi_3_Ti_7_O_x_-2	1.887	5.383

Nevertheless, the observed OER and ClER activities were incompletely justified with the areal capacitance and ohmic resistance, particularly for the unexpected elevated current generation from TiO_2_-L anode. A recent report presented evidences that the OER and ClER overpotential values of IrO_2_ could be reduced upon a few cycles of atomic layer deposition (ALD) of thin TiO_2_ layer by shifting surface charge density and overall M-O bond strength [21]. Underlying Ir^4+^ was speculated to be partly oxidized to result in sub-stoichiometric TiO_2−x_ outer layer, significantly elevating the electrical conductivity. Even for much thicker TiO_2_ coating (greater than1 μm) in this study, Ir ions might be diffused into the TiO_2_ matrix during the thermal annealing, leading to a vertical concentration gradient of Ir (in mixture with Ti). Effective ionic radius of Ir^4+^ (62.5 pm) is more similar with Ti^4+^ (60.5 pm) than Bi^3+^ (103 pm), supporting the observed enhancement only with the TiO_2_–L layer. For the TiO_2_–H heterojunction anode, more pronounced increase in ohmic resistance led to net reduction in anodic wave, as in the case of far thicker (~30 µm) TiO_2_ layers on IrTaO_y_ in our previous report [11].

### 3.3 Reactive chlorine generation by IrTaO_y_/(Bi_2_O_3_)_x_(TiO_2_)_1−x_ heterojunction anodes

**Fig. 3:**
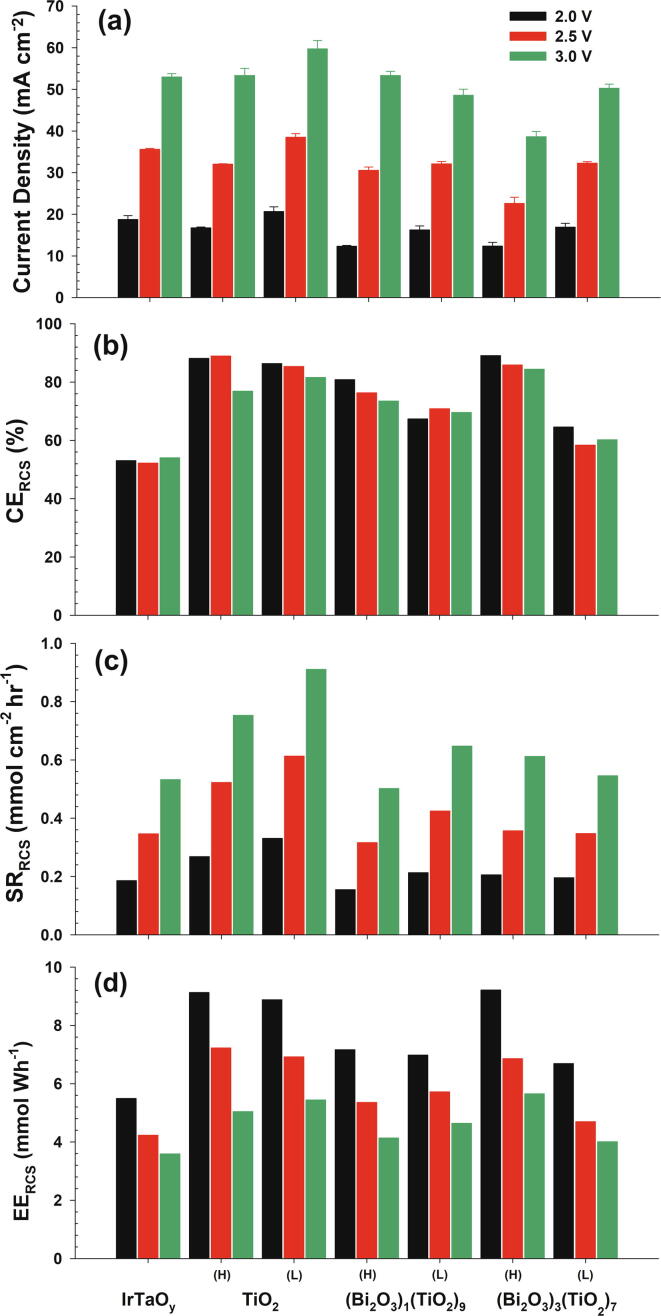
(a) Current density, (b) current efficiency, (c) specific rate and (d) energy efficiency of reactive chlorine species generation during potentiostatic electrolysis of 50 mM NaCl solutions (pH 7) with IrTaO_y_/(Bi_2_O_3_)_x_(TiO_2_)_1−x_ heterojunction anodes (x = 0, 0.1, and 0.3; mass loading = high (H) and low (L)); cathode: stainless steel, geometric surface area: 3 × 2 cm^2^, applied anodic potential: 2.0, 2.5, and 3.0 V NHE. Data for IrTaO_y_ anode are shown as references.

Reactive chlorine generation was comparatively evaluated in 50 mM NaCl solutions (Fig. 3) at variable applied anodic potentials (2.0, 2.5, and 3.0 V NHE). Throughout this study, the corresponding cell voltage and ohmic resistance (measured by current interruption at 200 mA) ranged 4–6 V and 3.3–5.6 Ω, respectively. The current density in potentiostatic condition (Fig. 3a) was in general agreement with the LSV shown in Fig. 2b. EE always decreased with the greater applied potential (cell voltage) due to the increment in ohmic drop, whereas CE varied insignificantly. Despite moderate variations in ohmic drop and iR-compensated anodic potential among anodes, the insignificant dependency of CE on the applied potential would allow a qualitative comparison. The CE and EE values of the control IrTaO_y_ electrode were averaged to 52% and 4.23 mmol Wh^−1^ at 2.5 V NHE, respectively, while the heterojunction layers substantially augmented the CE and EE for RCS generation (in Fig. 3b and d). Accordingly, despite the general reductions in the operational current density, heterojunction anodes marked greater specific RCS generation rates (Fig. 3c) than the control. The most remarkable enhancement was noted for TiO_2_-L with more than 70% increase in SR_RCS_, due to the elevated voltammetric response. Utilizing nanoparticle slurry precursor with relatively simple preparation procedure, these results outperformed our previous report on IrTaO_y_/Bi_x_Ti_1−x_O_z_ heterojunction anodes by casting aqueous Ti-glycolate solutions prepared by a peroxo-route [11].

It would be a rational postulation that the RCS generation through charge transfer between the physi-sorbed ∙OH and Cl^−^ (Eq. (5)) would be far more facile than oxygen atom transfer from higher oxide (Eq. (6)) [5]. In comparison, the >MO_x+1_ form would more preferentially mediate OER (Eq. (4)) than (>MO_x_ (∙OH), Eq. (3)) [10]. Therefore, the speciation of hydrous metal oxide under an anodic bias (MO_x_(∙OH) versus MO_x+1_) is expected to determine the selectivity between OER and ClER [22], [23]. More predominant steady-state surface concentration of >MO_x_(∙OH) would account for the overall enhancements of CE and EE for RCS generation on heterojunction architectures, as suggested previously [11], [13]. In order words, primary Ti^4+^ species on surface of heterojunction anodes would prevent transformation into the higher oxide, whereas the control IrTaO_y_ would favor >MO_x+1_ formation by Ir^4+^/Ir^6+^ transition.

Compared to IrTaO_y_/TiO_2_ anodes, on the other hand, Bi_2_O_3_ particles on surface generally lessened the CE_RCS_ and EE_RCS_, except for (Bi_2_O_3_)_3_(TiO_2_)_7_-H. Our previous report [11] proposed competitive roles of Bi mixing for ClER on the solution casted IrTaO_y_/Bi_x_Ti_1−x_O_z_ heterojunction anodes; accelerating interaction with Cl^−^ (*via* a positive shift in surface charge) but hampering ClER kinetics through the higher oxide formation (*via* Bi^3+^/Bi^5+^ redox transition). Accordingly, CE values of ClER were noted to be maximized at Bi fraction of 0.1–0.3 [11]. In this study, the former effect was insignificant, most presumably because of spatially separated TiO_2_ and Bi_2_O_3_ phase. Nevertheless, (Bi_2_O_3_)_3_(TiO_2_)_7_-H with the highest surface loading of Bi showed comparable CE and EE values with IrTaO_y_/TiO_2_ anodes.

Although the improved CE and EE of RCS generation on (Bi_2_O_3_)_3_(TiO_2_)_7_-H, limited stability in anodic environments was noted specifically due to the dissolution of Bi_2_O_3_ particles. Figure S6a illustrates the concentrations of Bi and Ti in electrolyte during an accelerated life test at 1000 mA cm^−2^ operation for the (Bi_2_O_3_)_3_(TiO_2_)_7_-H heterojunction anode. Bi_2_O_3_ particles were expected to maintain intrinsic properties so as to be vulnerable to anodic potential bias and local acidity from OER, as confirmed by a distinct accumulation of Bi in electrolyte. EDS mapping (Figure S6b-f) after the life test also located intensified signals of Ir on remains with dissolution/detachment of Bi_2_O_3_ particles. The concurrent rise of Ti concentration would be associated with detachment of TiO_2_ nanoparticles along with the collapse of particle aggregation. In summary, decoration of micron-sized Bi_2_O_3_ particles on the TiO_2_ heterojunction brought about limited or even adverse effects both for RCS generation and stability.

### 3.4 Reactive chlorine generation by IrTaO_y_/Bi_3_Ti_7_O_x_ heterojunction anodes

In order to address the drawbacks of Bi_2_O_3_ decoration, this study evaluated two different Bi_3_Ti_7_O_x_ heterojunction layers with more uniform doping of Bi into the TiO_2_ matrix. For Bi_3_Ti_7_O_x_-1 anode, titanium glycolate nano-particle slurry was prepared by polyol-mediated synthesis [14], to be mixed with a bismuth citrate solution (Bi:Ti = 3:7) overnight. During this process, Bi^3+^ was expected to be randomly substituted with Ti^4+^ in the polyol structure by ligand exchange to form a homogeneous solid mixture during the subsequent thermal decomposition. In addition, a well-established co-precipitation procedure for metal ion doped nano-sized TiO_2_ colloidal suspension [24] was employed for preparation of Bi_3_Ti_7_O_x_-2 anode. Under the same spraying procedure, the mass loading of Bi_3_Ti_7_O_x_-1 layer was far greater (3.20 mg cm^−2^) than Bi_3_Ti_7_O_x_-2 (0.342 mg cm^−2^) due to the different precursor viscosity (aqueous versus organic solvent).

**Fig. 4:**
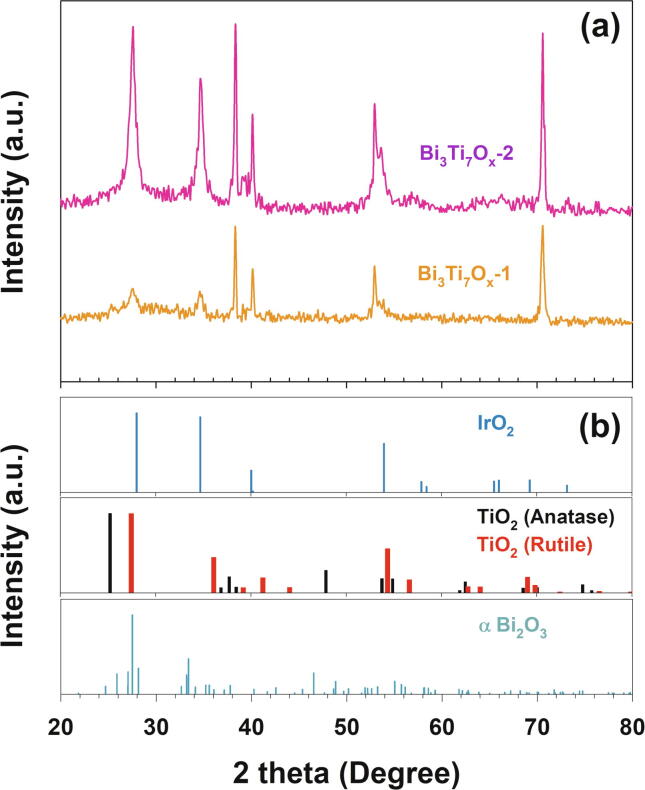
(a) X-ray diffraction patterns of (a) IrTaO_y_/(Bi_3_Ti_7_O_x_-1, 2) heterojunction anodes, referenced with (b) IrO_2_, TiO_2_ (anatase, rutile) and α-Bi_2_O_3_.

The SEM images on the horizontal surface of Bi_3_Ti_7_O_x_ anodes (Figure S7) showed rugged morphology without specific grains of Bi_2_O_3_. The outer film contained multiple cracks that have been typically found for annealed mixed metal oxide due to the variable thermal expansion coefficients [25]. In addition, XRD profiles (Fig. 4 and S8) for the both Bi_3_Ti_7_O_x_ anodes showed insignificant peaks relevant to Bi_2_O_3_. These observations would substantiate homogeneous mixed metal oxide formation by the modified preparation schemes. Besides reflections from Ti substrate, intense signals from rutile TiO_2_ dominated on Bi_3_Ti_7_O_x_-2. Insertion of Bi^3+^ into the TiO_2_ lattice would distort the crystalline structure to facilitate transformation of anatase to rutile at relatively low annealing temperature (450 °C) [16]. At superimposable locations, by far smaller peaks were noted on Bi_3_Ti_7_O_x_-1, suggesting predominant amorphous phase. Evidences were presented that polyol-mediated Ti-glycolate could remain amorphous up to 500 °C [14].

**Fig. 5:**
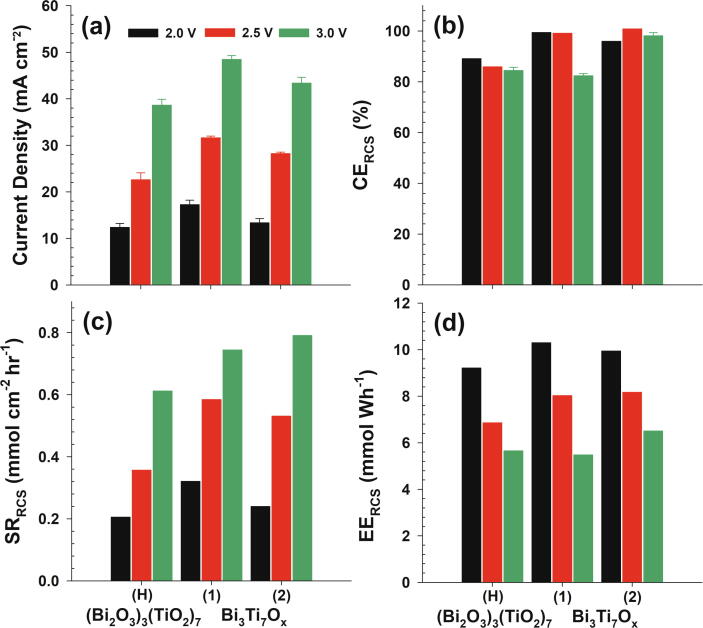
(a) Current density, (b) current efficiency, (c) specific rate and (d) energy efficiency of reactive chlorine species generation during potentiostatic electrolysis of 50 mM NaCl solutions (pH 7) with IrTaO_y_/(Bi_3_Ti_7_O_x_-1, 2) heterojunction anodes; cathode: stainless steel, geometric surface area: 3 × 2 cm^2^, applied anodic potential: 2.0, 2.5, and 3.0 V NHE. Data for IrTaO_y_/(Bi_2_O_3_)_3_(TiO_2_)_7_-H anode are shown as references.

CV presented in Figure S9a estimated areal capacitance to be 14.91 mF cm^−2^ and 21.96 mF cm^−2^ for Bi_3_Ti_7_O_x_-1 and −2, respectively. Moderately lower capacitance of Bi_3_Ti_7_O_x_-1 would be associated with the greater mass loading of outer layer and film resistance. During the LSV in 50 mM NaCl solutions (Figure S9b), anodic current waves on Bi_3_Ti_7_O_x_ anodes outperformed (Bi_2_O_3_)_3_(TiO_2_)_7_–H anodes and the voltammogram of Bi_3_Ti_7_O_x_-1 was comparable with TiO_2_-H. Despite the lack of conclusive evidence, coordinately unsaturated active sites in amorphous structure (Bi_3_Ti_7_O_x_-1) could moderately facilitate the kinetics of OER and ClER. Fig. 5 shows that both Bi_3_Ti_7_O_x_ anodes marked CE_RCS_ near unity (93.6% and 98.3%, on average). Consequently, the IrTaO_y_/Bi_3_Ti_7_O_x_ heterojunction anodes marked the highest EE_RCS_ (8.02–8.16 mmol Wh^−1^ at 2.5 V NHE) among the anodes interrogated in this study. These electrodes even outperformed a commercial Ir based DSA (De Nora) which showed lower values of CE_RCS_ (40.9% on average) and EE_RCS_ (3.1 mmol Wh^−1^ at 2.5 V NHE) as shown in Figure S10. These results demonstrated that even with the analogous composition, mixing level of constituents could significantly affect the electronic and electrostatic properties. In particular, relatively homogenous intercalation of Bi component might avoid the ohmic resistance across multiple junctions, while increase the point of zero charge for enhanced electro-sorption of Cl^−^. Furthermore, accelerated life test at 1000 mA cm^−2^ indicated insignificant dissolution of Bi and Ti during operation for 30 h (data not shown).

In order to evaluate the effects of ion migration upon the potential bias (increasing [Cl^−^] in the anode vicinity), the RCS generation experiments were performed in 50 mM NaCl solutions with or without another electrolyte (50 mM NaClO_4_) under a galvanostatic condition (Figure S11). The measured values of SR_RCS_, CE_RCS_, and EE_RCS_ in the mixed solutions were 82, 86, and 97% of those in NaCl solutions, respectively. Moderate reductions could be associated with the competing ion migration and lower potential bias in the presence of other electrolyte, which would be a consideration to understand the performance in real (waste)water matrix. On the other hand, one may argue that the elevated CE_RCS_ by the heterojunction architecture would be ascribed to the reduced current density, since an increasing current could invigorate OER only when ClER is rate-limited by diffusion of Cl^−^. However, the operational current density in this study would be lower than the limiting current density for ClER, as corroborated by Figure S12. The observed CE_RCS_ values showed negligible correlation with the current density. Therefore, the shift in ClER selectivity should be understood by speciation of surface intermediates with variable reactivity with Cl^−^ (*vide infra*).

### 3.5 Formate ion degradation versus RCS generation to assess intermediate speciation

In this study, the hydroxyl radical (∙OH) on anode surface could be a nonselective oxidant to be utilized for Cl^−^ oxidation to RCS. Due to the short lifetime (10^−6^ − 10^−3^ s), free or bound ∙OH has been quantified using an array of probe compounds such as salicylic acid, benzoic acid, coumarin, benzoquinone, and RNO (*p*-nitrosodimethylaniline) [26]. Formate ion could be another surface ∙OH prove due to superb reactivity with ∙OH [27], simple quantification method using IC, and relatively low molecular weight to avoid diffusion limitation.

In order to further interrogate the speciation between MO_x_(∙OH) and MO_x+1_ and their roles on RCS generation, potentiostatic (*E_a_* = 2.0 or 2.5 V NHE) formate ion degradation experiments (initial concentration = 50 mM) were performed using the series of (Bi_2_O_3_)_x_(TiO_2_)_1−x_ and Bi_3_Ti_7_O_x_ heterojunction anodes (Figure S13). We limited the applied anodic potential up to 2.5 V NHE due to slightly lower electrical conductivity of 50 mM NaCOOH solutions (4.3 mS cm^−1^) than 50 mM NaCl solutions (5.4 mS cm^−1^). The differences in ohmic drop between two solutions were estimated to be 0.16 V at maximum. The reaction between HCOO^–^ and > MO_x_(·OH) could be approximated to pseudo-first-order owing to the facile kinetics (Eq. (10)).

(10) >MO_x_(∙OH) + HCOO^–^ → >MO_x_ + COO^–^∙ + H_2_O

**Fig. 6:**
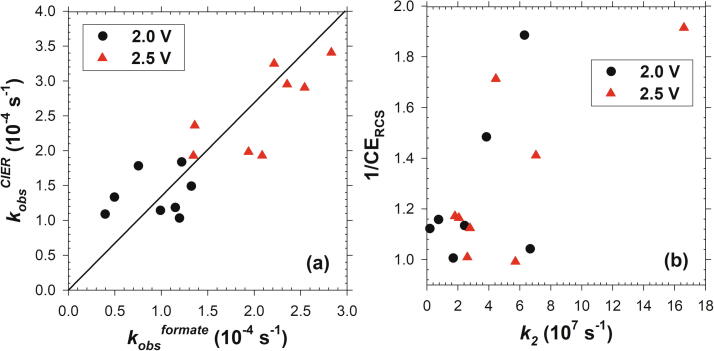
Correlations between (a) rate constants of ClER (*k_obs_^ClER^*) and formate degradation (*k_obs_^formate^*), together with (b) current efficiency of reactive chlorine generation (CE_RCS_) and transition rate constant (*k_2_*) from MO_x_(∙OH) to MO_x+1_.

Fig. 6a illustrates the observed pseudo-first-order rate constants of formate decay (*k_obs_^formate^*) in relation with the pseudo-first order rate constants of RCS generation (*k_obs_^ClER^*) that were estimated from the SR_RCS_ (Figs. 3c and 5c) and initial chloride concentration (*k_obs_^ClER^* = SR_RCS_ *A* /*V* [Cl^−^]_0_). The *k_obs_^ClER^* was observed to be linearly correlated with *k_obs_^formate^* and, more importantly, the slope of the regressed line was close to the ratio of bimolecular rate constant of ∙OH with Cl^−^ (*k_∙OH_^chloride^* = 4.3 × 10^9^ M^−1^ s^−1^) to that with HCOO^–^ (*k_∙OH_^formate^* = 3.2 × 10^9^ M^−1^ s^−1^). These findings strongly evinced that MO_x_(∙OH) would be the exclusive intermediate for the RCS generation (Eq. (5)), while MO_x+1_ driven ClER (Eq. (6)) would play a minor role for our heterojunction anodes. In spite of previous reports [28] on HCOOH degradation by IrO_2_/IrO_3_ couple in acid (pH 0), oxygen atom transfer from MO_x+1_ to HCOO^–^ could be excluded in the current experimental conditions. Ozone, as a presumed analog of the higher oxide, is known to undergo sluggish kinetics both with Cl^−^ (<3 × 10^−3^ M^−1^ s^−1^) and HCOO^–^ (1 × 10^2^ M^−1^ s^−1^) [29], [30].

Provided that free and bound ∙OH react with HCOO^–^ at analogous bimolecular rate constants, the steady-state concentration of surface bound ∙OH ([>MO_x_(∙OH)]_SS_) could be estimated from *k^obs^_formate_* using eq. (11).

(11) [>MO_x_(∙OH)]_SS_ = *k_obs_^formate^* (s^−1^) *V* / (*A k_∙OH_^formate^*)

As shown in Figure S14a, the [>MO_x_(∙OH)]_SS_ estimates were in similar order with our previous study [11]. Eq. (12) given by a pseudo-steady-state approximation further estimated the rate constant for Eq. (2) (*k_2_*), assuming that >MO_x_(∙OH) and >MO_x+1_ are exclusively responsible to the formate ion oxidation and OER, respectively.

(12) $$k_{2}=\frac{CE_{\mathrm{OER}}I}{2F}\times \frac{1}{A{\left[>MO_{x}\left(\cdot OH\right)\right]}_{\mathrm{SS}}}$$

where CE_OER_ is the CE of OER calculated from the charge balance (1 – CE of formate ion degradation). Figure S14b substantiated that the outer heterojunction layers significantly lowered *k_2_* values of IrTaO_y_. Under further assumption that transition from >MO_x_(∙OH) to >MO_x+1_ (Eq. (2)) is limiting the OER rate, CE_RCS_ would be determined by the ratio of *k_2_* to *k_obs_^chloride^*. Despite the apparent inverse relation between *k_2_* and CE_RCS_ noted in Fig. 6b, relatively weak correlation suggested the liberation of O_2_ from >MO_x+1_ (Eq. (4)) could also determine the OER rate depending on the outer layer composition. For example, a strong binding of O on Bi might allow the ClER to overwhelm OER on the Bi_3_Ti_7_O_x_-2 anode with relatively high *k_2_* value.

## 4 Conclusions

This study prepared Ir_7_Ta_3_O_y_ DSAs with outer heterojunction layers based on mixed Bi and Ti oxide to compare the RCS generation efficiency during competitive OER and ClER in dilute (50 mM) NaCl solutions. Crack free, anatase dominant TiO_2_ layers prepared from ball-milled P60 nanoparticles brought about significantly enhanced SR_RCS_, CE_RCS_, and EE_RCS_, while TiO_2_-L even elevated the anodic current wave possibly owing to a tuned overall M-O bond strength. However, decoration of Bi_2_O_3_ particles on the outer TiO_2_ films ((Bi_2_O_3_)_x_(TiO_2_)_1−x_) gave limited or adverse effects on voltammetric response, RCS generation, and stability presumably because of multiple junction formation and specially separated Bi_2_O_3_ phase. To this end, Bi_3_Ti_7_O_x_ heterojunction layers with elevated mixing level of Bi marked CE_RCS_ values near unity, by an effective increase in the point of zero charge. Consequently, the highest SR_RCS_ and EE_RCS_ were noted by Ir_7_Ta_3_O_y_/TiO_2_-L and Ir_7_Ta_3_O_y_/Bi_3_Ti_7_O_x_, respectively. Parallel formate ion degradation experiments revealed that the RCS generation would be exclusively mediated by >MO_x_(·OH) whose further transition to >MO_x+1_ was proven to be retarded by the heterojunction layer. Our heterojunction anodes with simplified preparation procedure would allow an energy-efficient electrochemical oxidation processes for water disinfection and high salinity wastewater treatment through the enhanced chlorine evolution. Improvements in the industrial chlorine generation (chlor-alkali process) are also expected with reduced power input and operational cost. Further study should tackle the effects of outer layer TiO_2_ loading (thickness) on film resistance and CE_RCS_, as well as stability of the Bi-doped TiO_2_ layers in more systematic manners based on periodic CE_RCS_ measurements during accelerated life test.
