# Supplementary material for: Enhanced chlorine evolution from dimensionally stable anode by heterojunction with Ti and Bi based mixed metal oxide layers prepared from nanoparticle slurry
Source: J Catal. 2020 Sep;389:1–8. doi: 10.1016/j.jcat.2020.04.009 (PMC7539370; doi:10.1016/j.jcat.2020.04.009)
Supplement: Supplementary data 1 [file mmc1.docx]

**Supporting Information**

**Enhanced chlorine evolution from dimensionally stable anode by heterojunction with Ti and Bi based mixed metal oxide layers prepared from nanoparticle slurry**

Sukhwa Hong^a^, Tai-kyu Lee^b^, Michael R. Hoffmann^c^, and Kangwoo Cho^a,*^

*^a^ Division of Environmental Science and Engineering, Pohang University of Science and Technology (POSTECH), Pohang 790-784, Korea.*

*^b^ Nanopac Co., Ltd. 673 Hwasan-Ri, Cheoin-Gu, Yongin-Si, Gyeonggi-Do, Korea*

*^c^ Linde+Robinson Laboratories, California Institute of Technology, 1200 E. California Blvd., Pasadena, California 91125, USA.*

Submitted to

***Journal of Catalysis***

*Corresponding Author (K. Cho)
phone: +82 54 279 2289; fax: +82 54 279 8299; email: kwcho1982@postech.ac.kr

**Effects of Precursor Solvent on the IrTaO_y_ Layer**

Figure S1 shows SEM images on the horizontal surface of IrTaO_y_ anodes synthesized with aqueous (4 N HCl) and organic solvent (1:1 ethanol/isopropanol), respectively. IrTaO_y_ layer prepared with the organic solvent showed far more uniform surface topography with smaller surface cracks and curvature. IrTaO_y_ based anode prepared by organic solvent had wider areal capacitance than aqueous based electrode (Figure S2a) and also showed a more stable electrochemical response in linear sweep voltammetry (Figure S2b). Nevertheless, comparable RCS generation rates were noted in 50 mM NaCl solutions (Figure S2c).


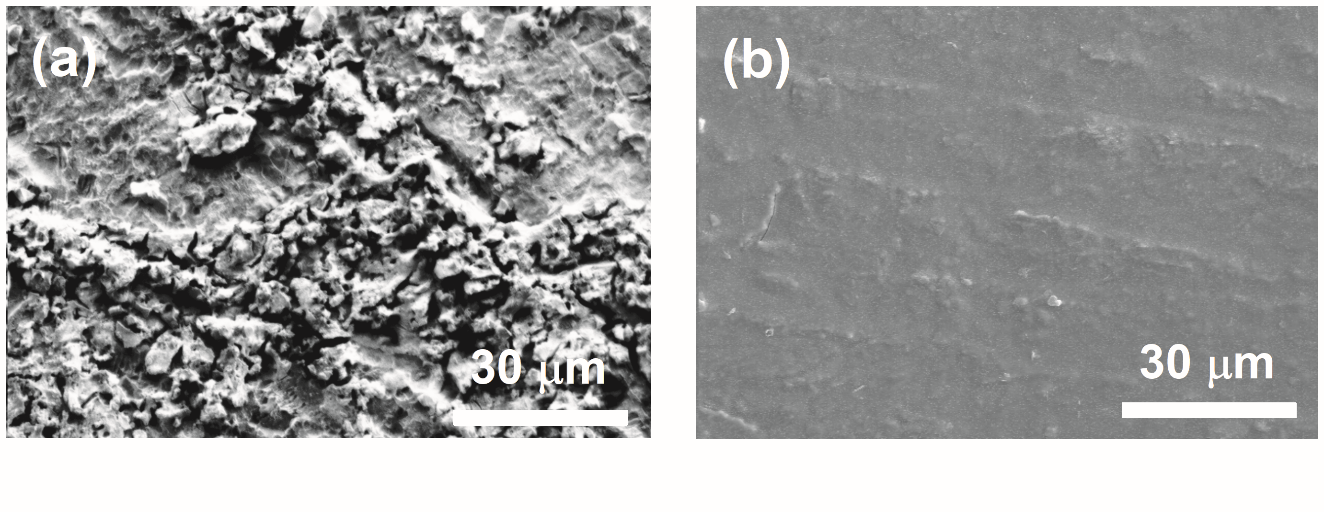


**Figure S1.** Scanning electron microscopy images on horizontal surface of Ir_7_Ta_3_O_y_ anodes using aqueous (a) and organic solvent (b).

**
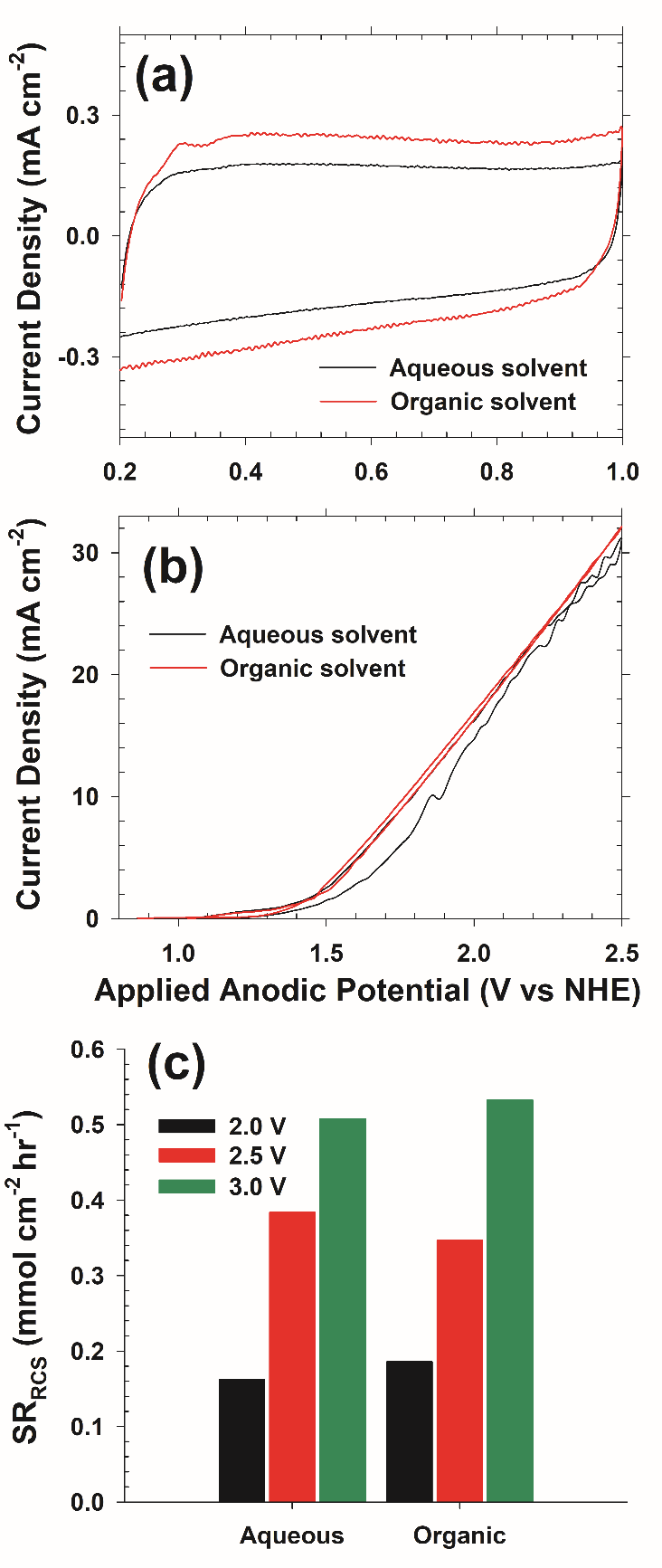
**

**Figure S2.** (a) Cyclic voltammograms (scan range: 0.2 - 1.0 V, scan rate: 20 mV s^−1^), (b) linear sweep voltammograms (scan range: 0.8 - 2.0 V, scan rate: 5 mV s^−1^), and specific rate of reactive chlorine generation by Ir_7_Ta_3_O_y_ anodes using aqueous and organic solvent.; electrolyte: 50 mM NaCl (pH 7), cathode: stainless steel, geometric surface area: 3 × 2 cm^2^.

**
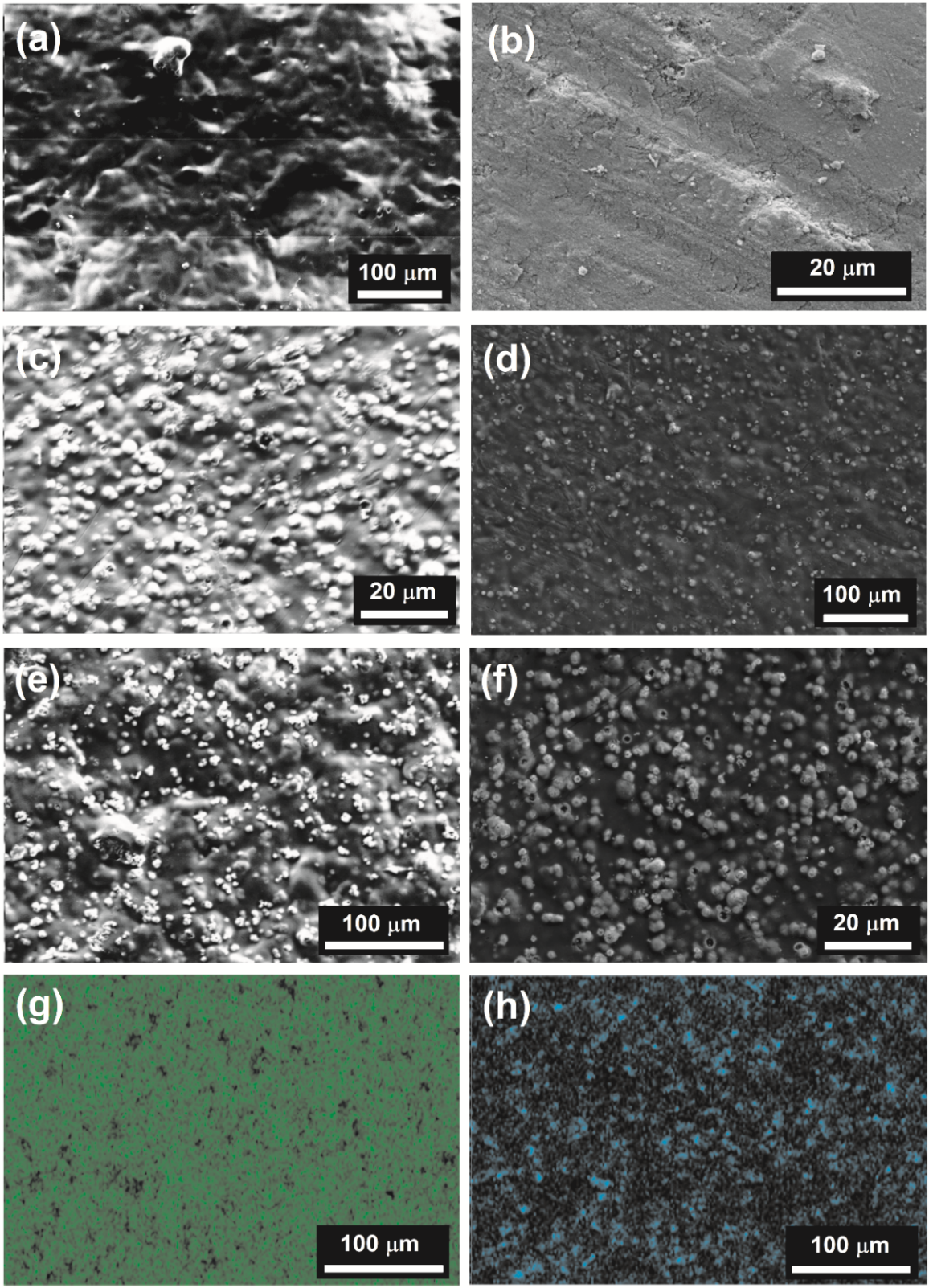
**

**Figure S3.** Scanning electron microscopy images on horizontal surface of IrTaO_y_/(Bi_2_O_3_)_x_(TiO_2_)_1-x_ heterojunction anodes; (a) TiO_2_-H, (b) TiO_2_-L, (c) (Bi_2_O_3_)_1_(TiO_2_)_9_-H, (d) (Bi_2_O_3_)_1_(TiO_2_)_9_-L, (e) (Bi_2_O_3_)_3_(TiO_2_)_7_-H, and (f) (Bi_2_O_3_)_3_(TiO_2_)_7_-L. Energy dispersive spectroscopy mapping of (g) Ti and (h) Bi are shown for (Bi_2_O_3_)_3_(TiO_2_)_7_-H. H and L denote high and low loading, respectively.

**
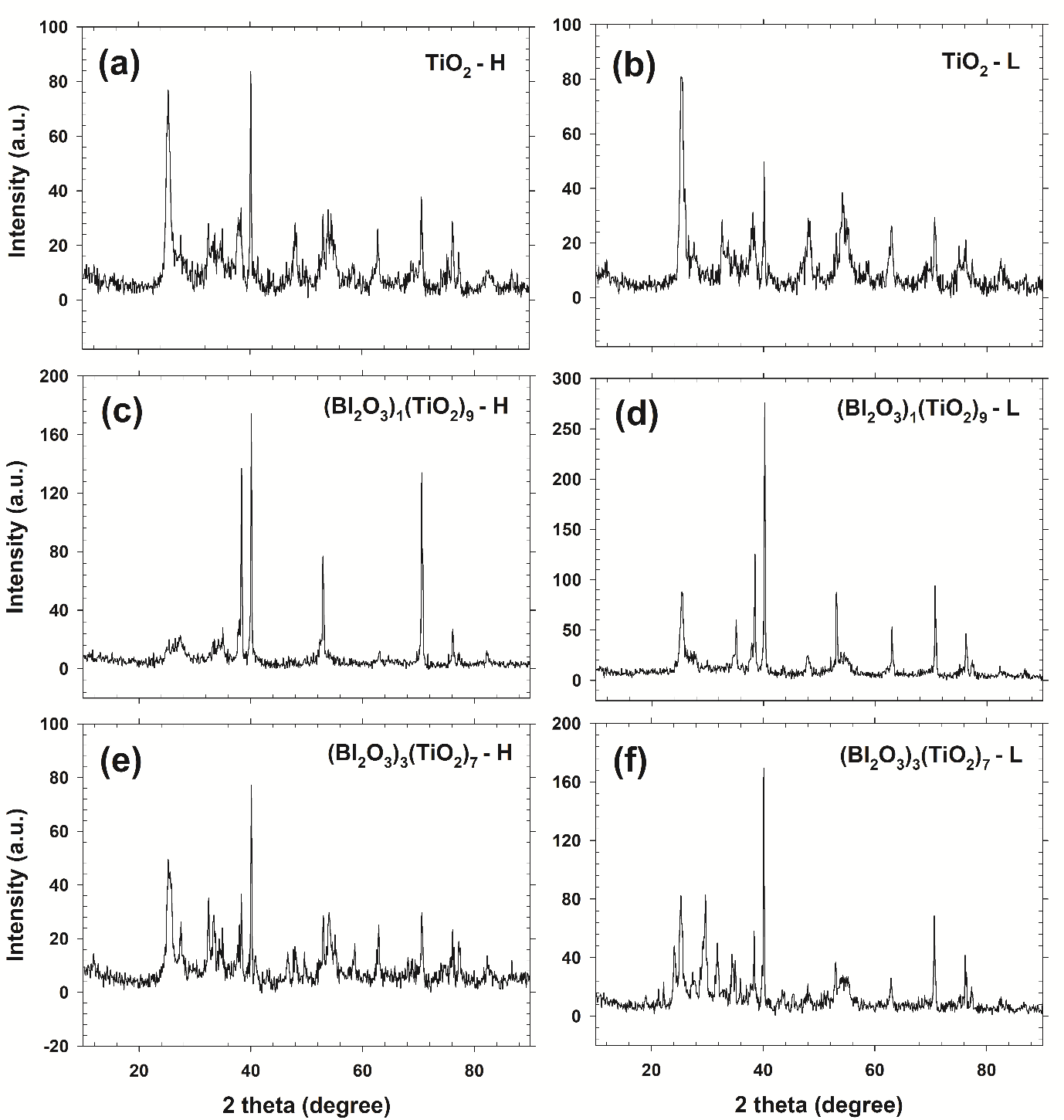
**

**Figure S4.** X-ray diffraction patterns of IrTaO_y_/(Bi_2_O_3_)_x_(TiO_2_)_1-x_ heterojunction anodes (x=0, 1, and 3; mass loading= high (H) and low (L)).

**
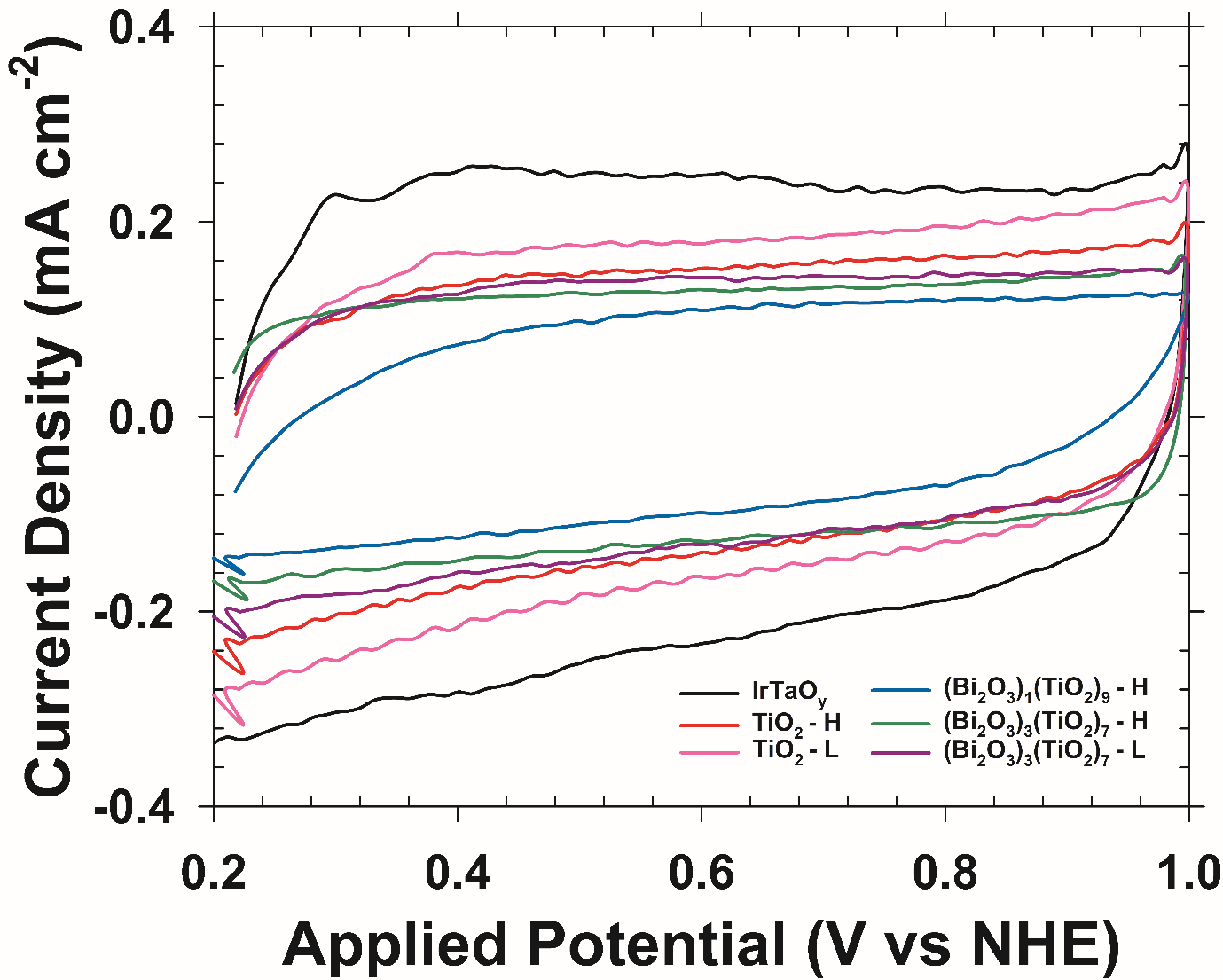
**

**Figure S5.** Cyclic voltammograms (scan range: 0.2 − 1.0 V, scan rate: 20 mV s^−1^) of IrTaO_y_/(Bi_2_O_3_)_x_(TiO_2_)_1-x_ heterojunction anodes (x=0, 0.1, and 0.3, mass loading= high (H) and low (L)); electrolyte: 50 mM NaCl (pH 7), cathode: stainless steel, geometric surface area: 3 × 2 cm^2^. Data for Ir_7_Ta_3_O_y_ anode are shown as references.

.

**
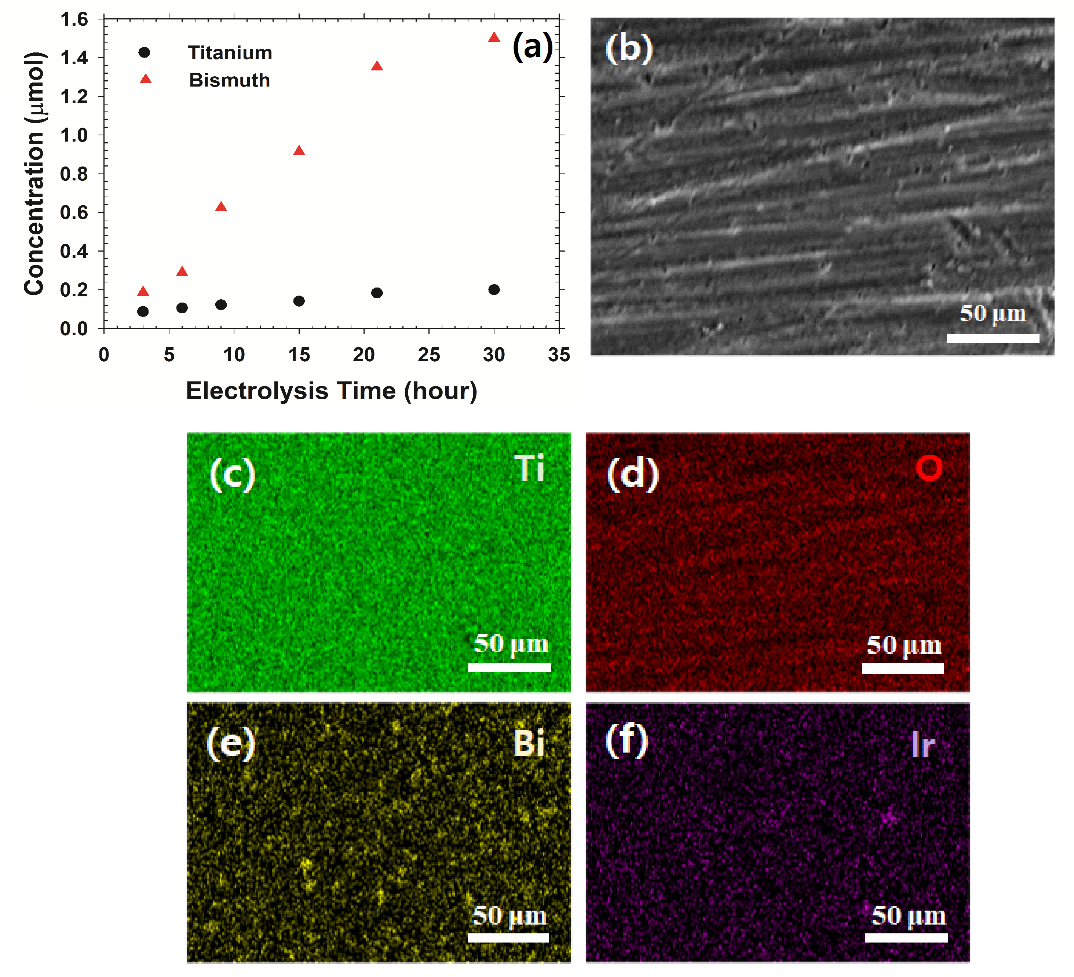
**

**Figure S6.** (a) Cumulative molar amounts of dissociated Bi and Ti in electrolyte during galvanostatic accelerated life test at 1 A cm^−2^ together with (b) scanning electron microscopy image and (c-f) energy dispersive spectroscopy mapping after the test for for IrTaO_y_/(Bi_2_O_3_)_3_(TiO_2_)_7_-H heterojunction anode; electrolyte: 1 M NaClO_4_ (pH: 7), cathode: stainless steel, geometric surface area: 2 × 0.5 cm^2^


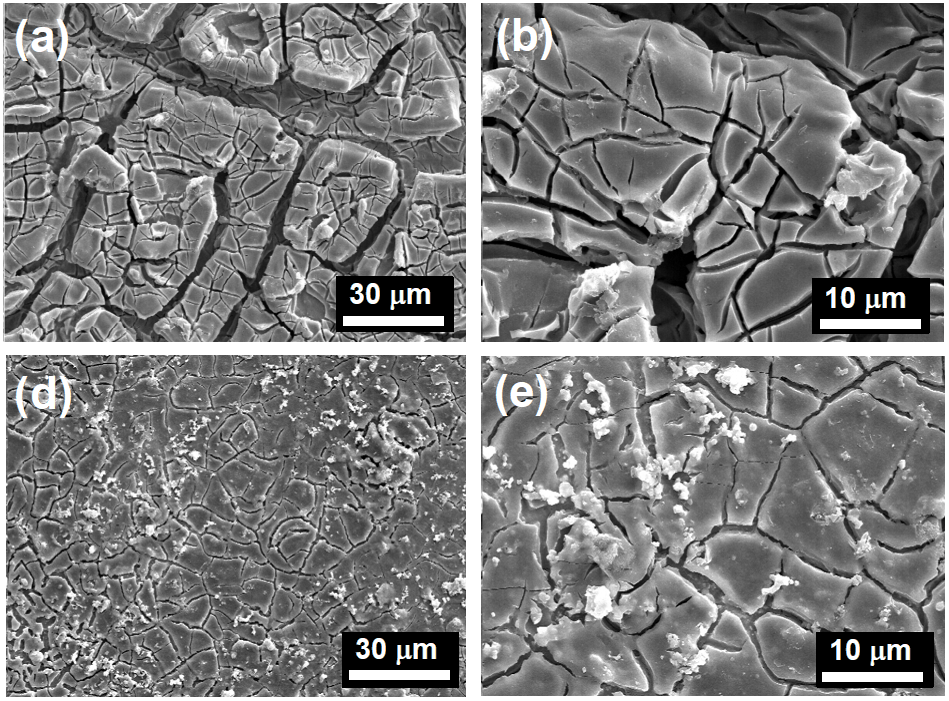


**Figure S7.** Scanning electron microscopy images on horizontal surface of IrTaO_y_ with outer layer of (a) Bi_3_Ti_7_O_x_-1 and (b) Bi_3_Ti_7_O_x_-2.

**
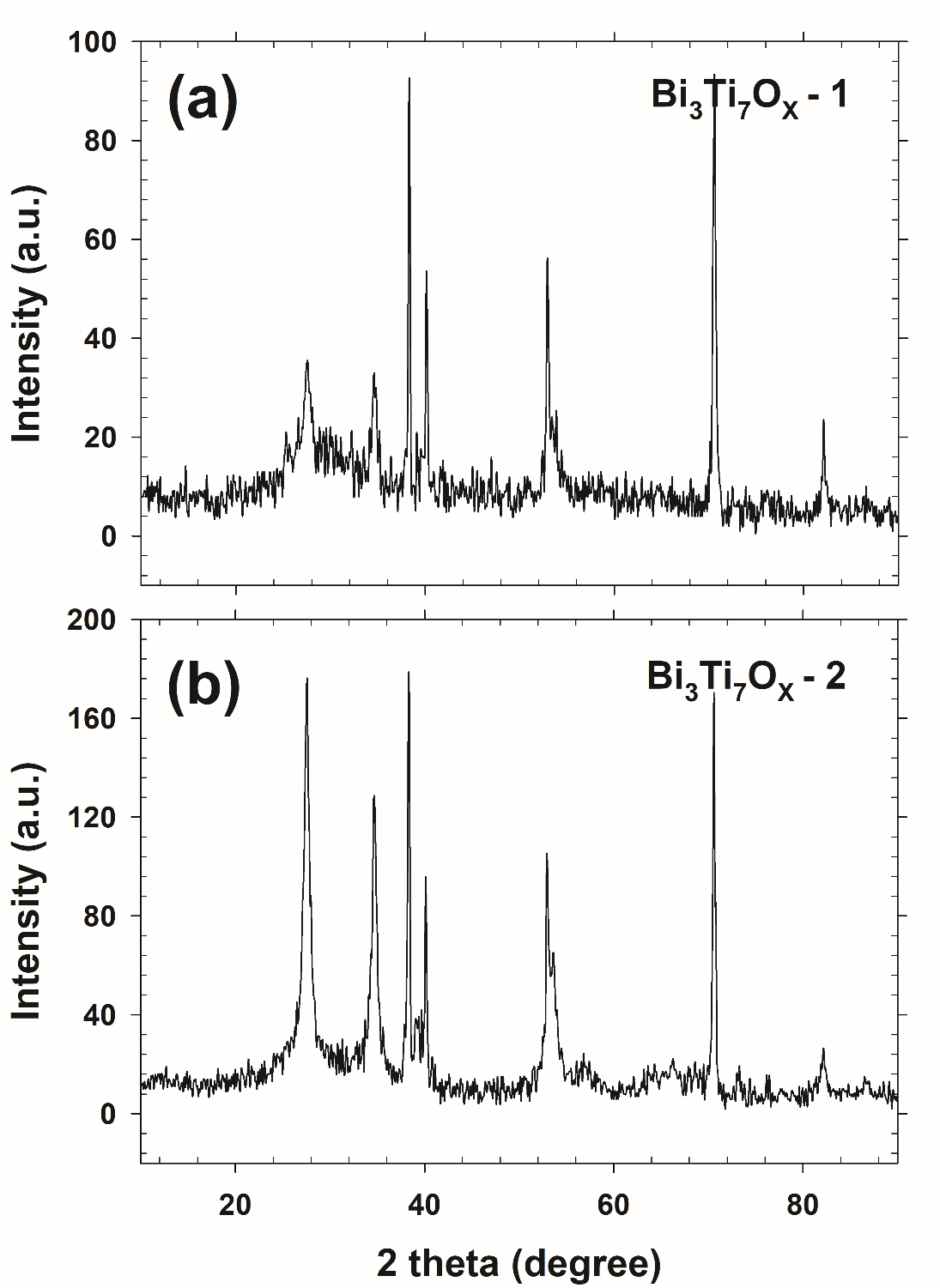
**

**Figure S8.** X-ray diffraction patterns of IrTaO_y_/(Bi_3_Ti_7_O_x_-1, 2) heterojunction anodes.

**
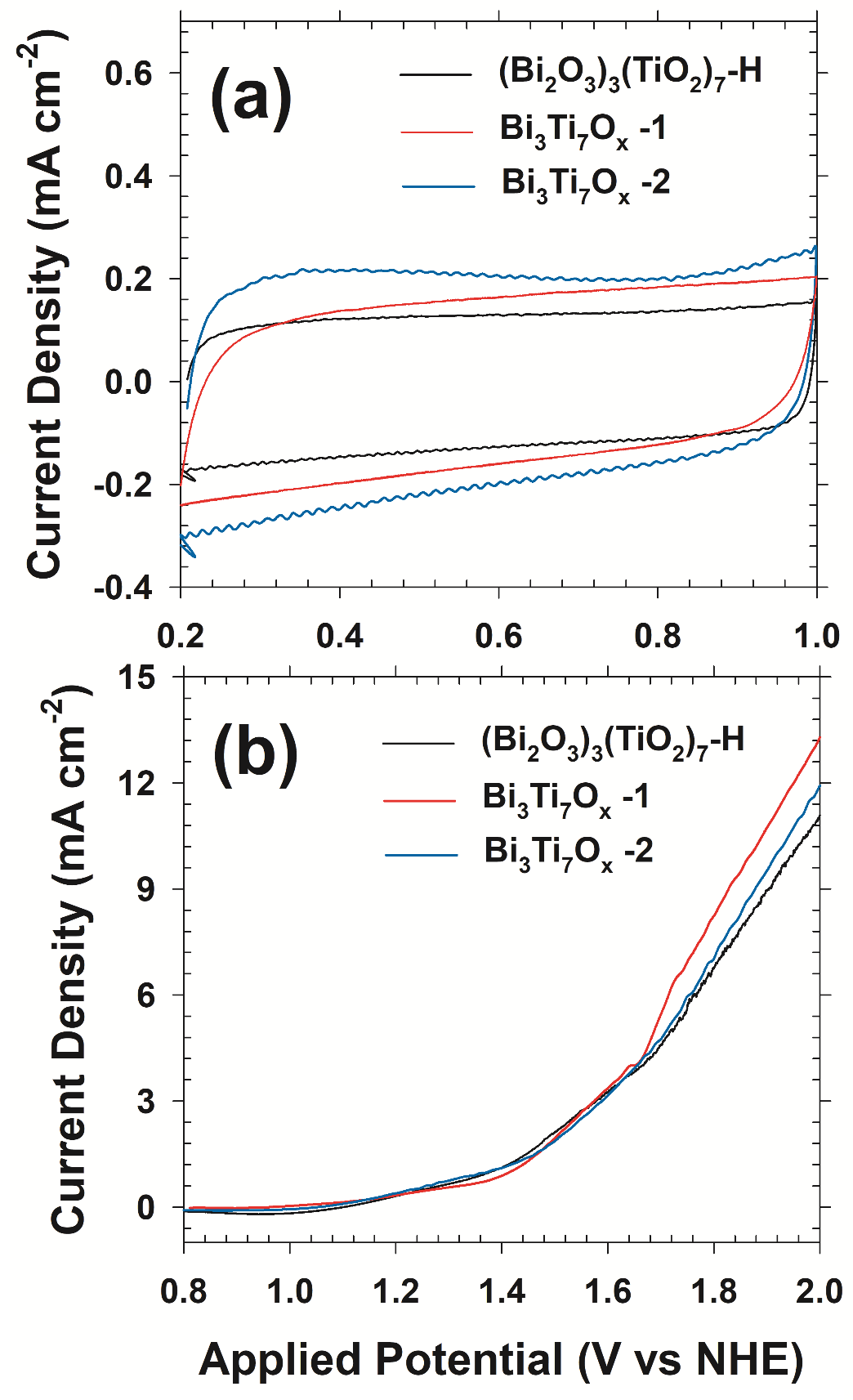
**

**Figure S9.** (a) Cyclic voltammograms (scan range: 0.2 − 1.0 V, scan rate: 20 mV s^−1^) and (b) linear sweep voltammograms (scan range: 0.8 - 2.0 V, scan rate: 5 mV s^−1^) of IrTaO_y_/(Bi_3_Ti_7_O_x_-1, 2) heterojunction anodes; electrolyte: 50 mM NaCl (pH 7), cathode: stainless steel, geometric surface area: 3 × 2 cm^2^. Data for IrTaO_y_/(Bi_2_O_3_)_3_(TiO_2_)_7_-H anode are shown as references.

**
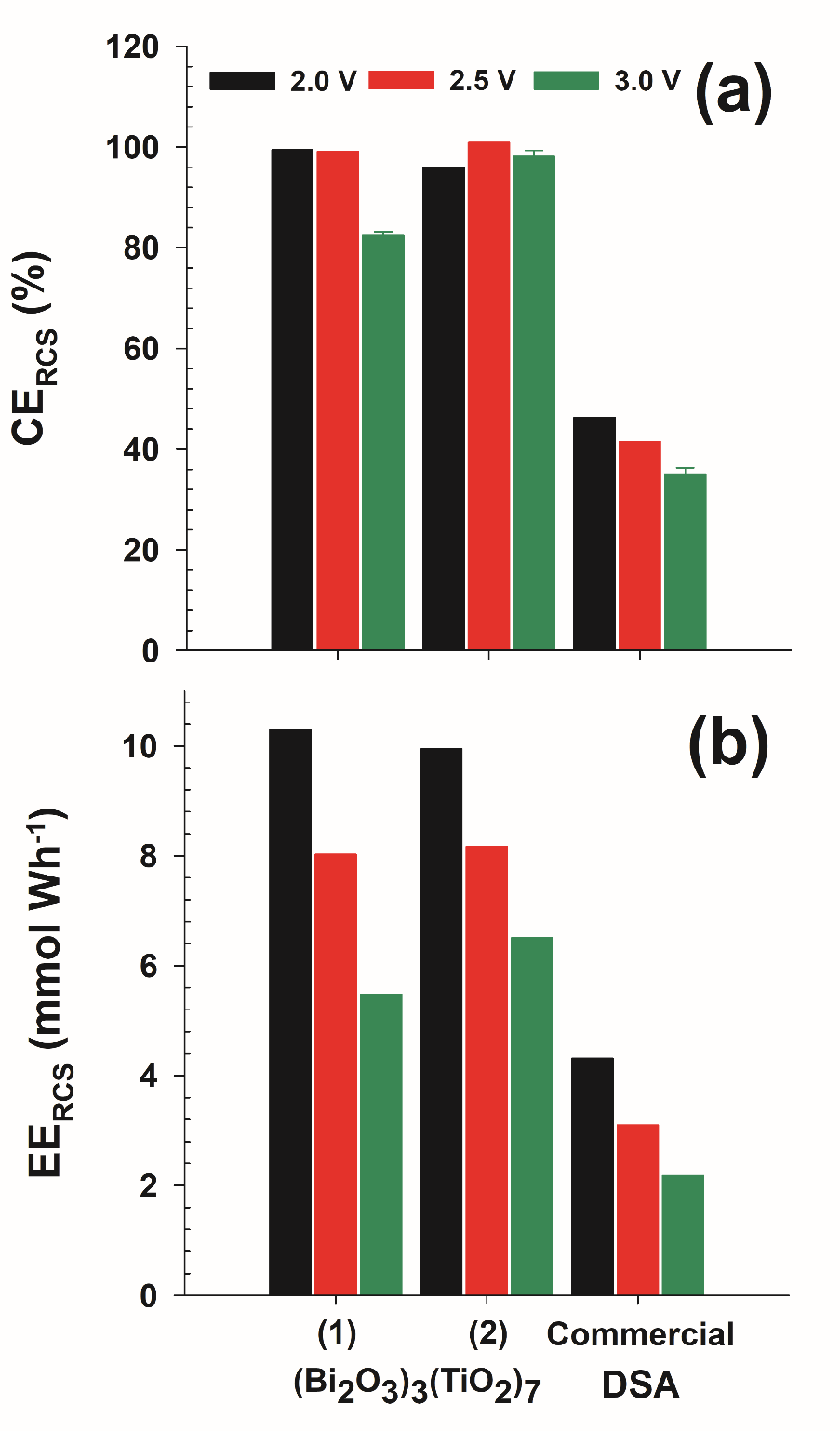
Figure S10.** (a) Current efficiency and (b) energy efficiency of reactive chlorine species generation during the potentiostatic electrolysis for IrTaO_y_/(Bi_3_Ti_7_O_x_-1, 2) heterojunction anodes and a commercial electrode (De Nora, Ir based DSA); electrolyte: 50 mM NaCl (pH: 7), cathode: stainless steel, geometric surface area: 3 × 2 cm^2^, applied anodic potential: 2.0, 2.5, and 3.0 V.

**
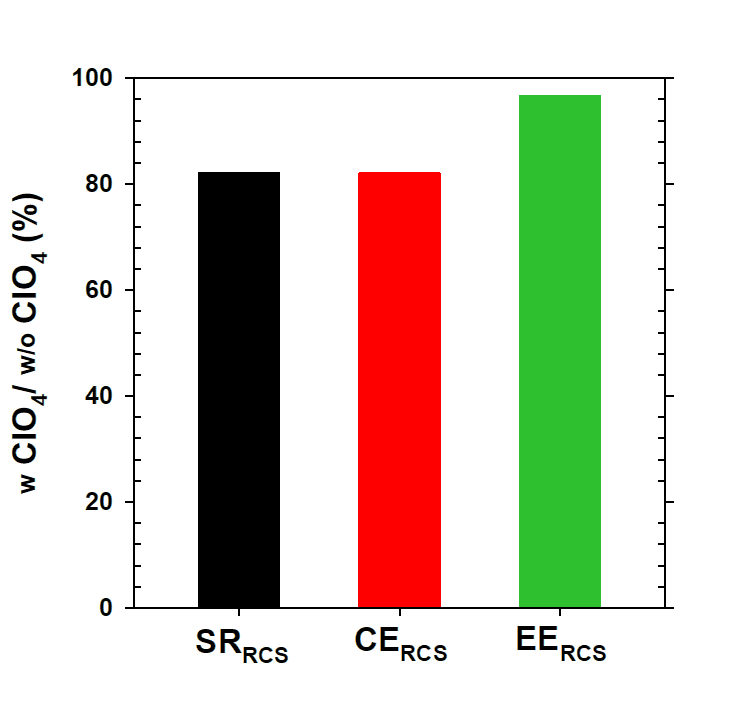
Figure S11.** Comparison of SR_RCS_, CE_RCS_, and EE_RCS_ values between different electrolyte solutions during galvanostatic electrolysis; electrolyte: 50 mM NaCl with/without 50 mM NaClO_4,_ cathode: stainless steel, geometric surface area: 3 × 2 cm^2^, applied current density: 20 mA cm^-2^.


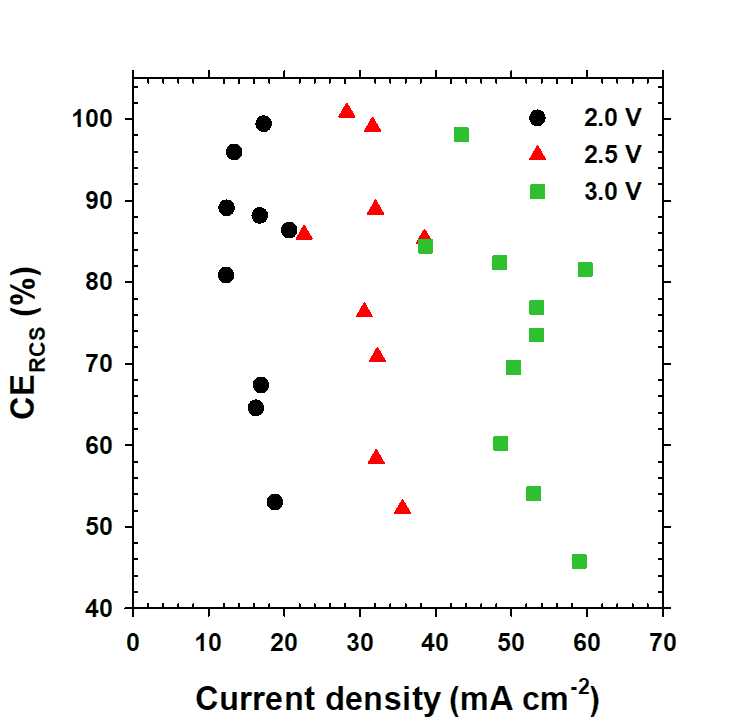


**Figure S12.** Correlation between CE_RCS_ and current density for all electrodes interrogated in this study; electrolyte: 50 mM NaCl (pH: 7), cathode: stainless steel, geometric surface area: 3 × 2 cm^2^, applied anodic potential: 2.0, 2.5, and 3.0 V.


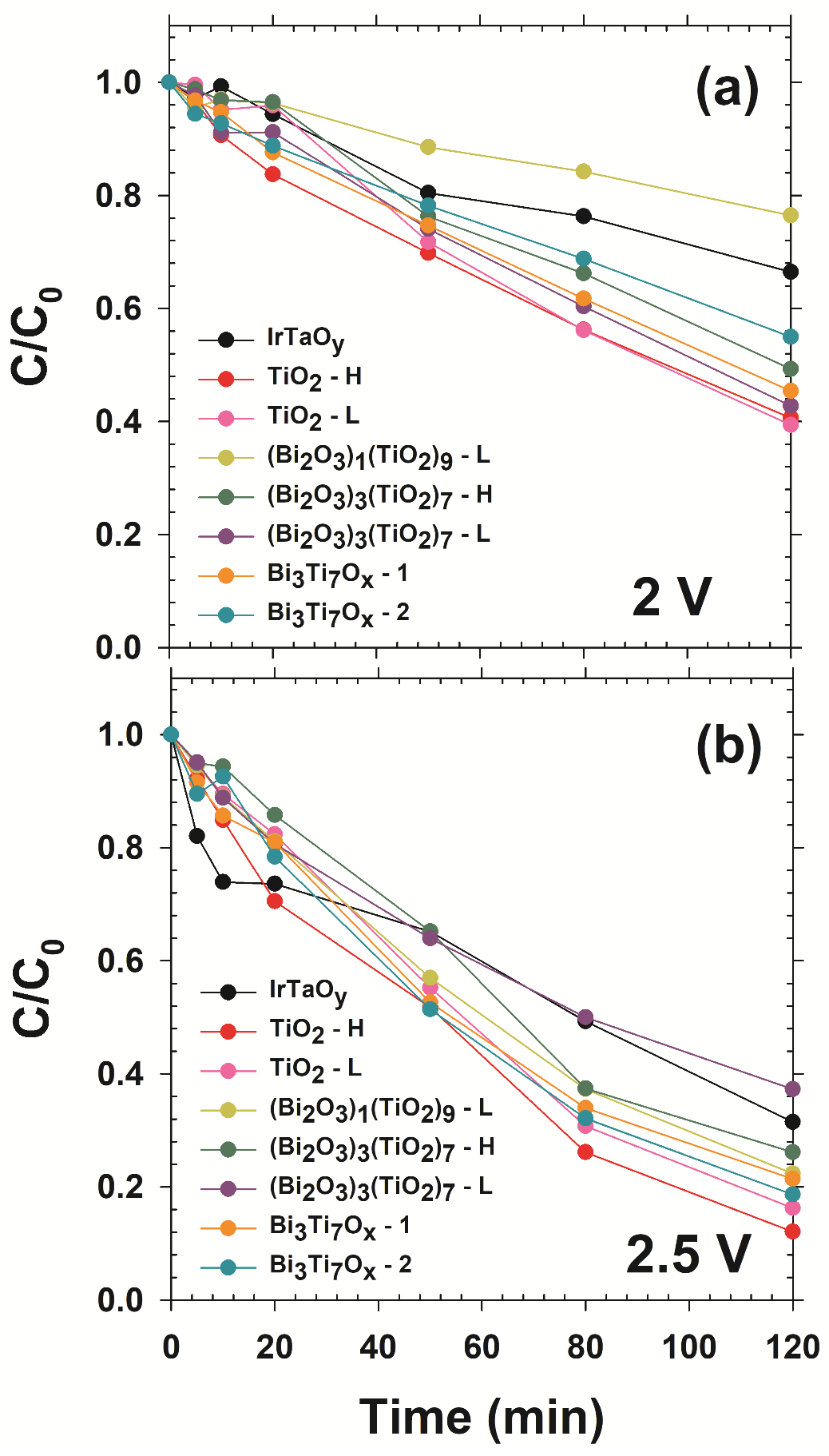


**Figure S13.** Formate ion degradation under potentiostatic electrolysis of HCOONa solutions at (a) 2 V NHE and (b) 2.5 V NHE; cathode: stainless steel, geometric surface area: 3 × 2 cm^2^,


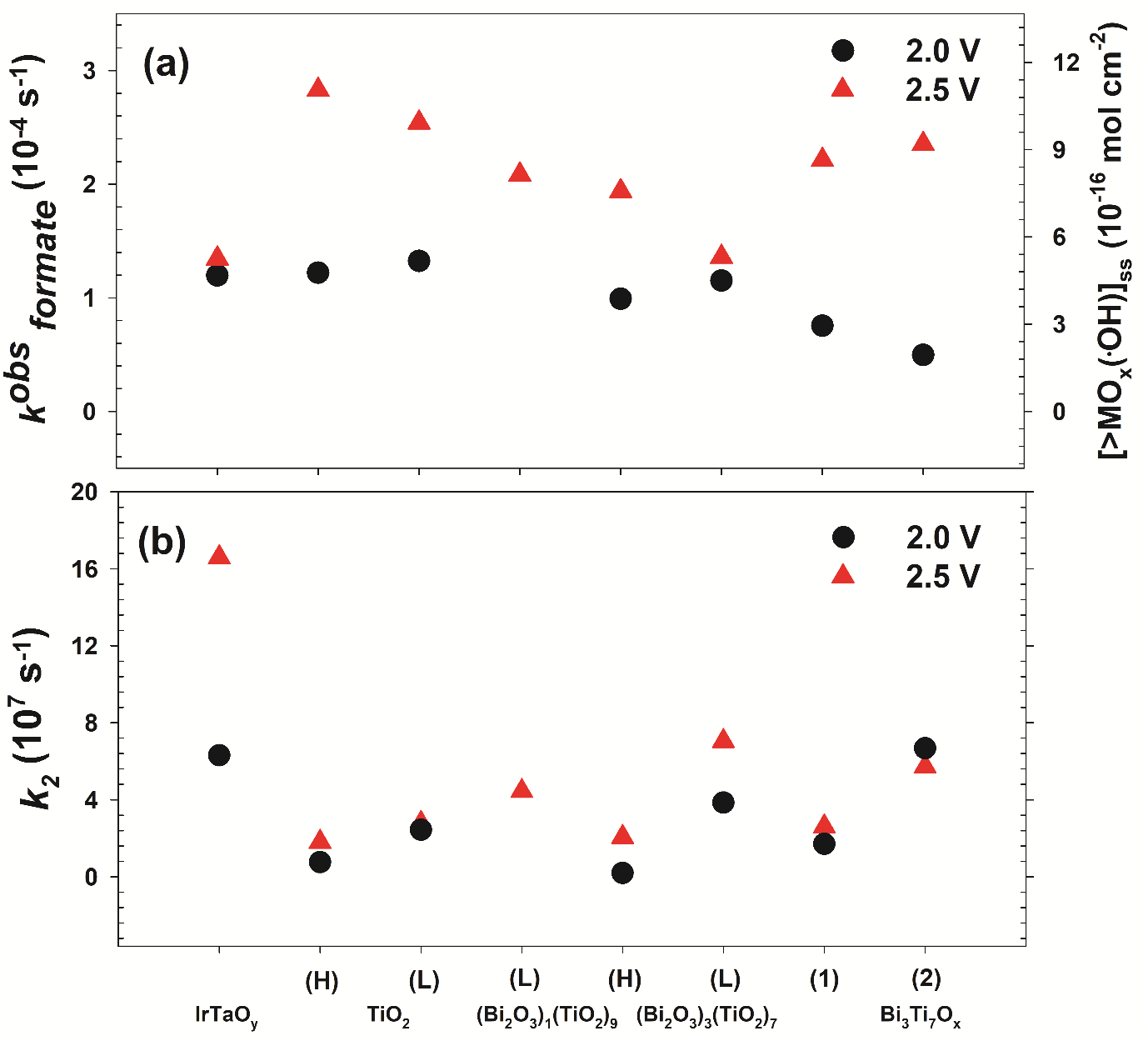


**Figure S14.** (a) Observed pseudo-first-order rate constant of formate ion degradation and steady-state concentration of surface hydroxyl radical together with (b) transition rate constant from MO_x_(∙OH) to MO_x+1_ (*k_2_*) estimated from data in Figure S10.
